# Analgesic regimens administered to older adults receiving skilled nursing facility care following hip fracture: a proof-of-concept federated analysis

**DOI:** 10.1186/s12877-024-05486-0

**Published:** 2024-10-30

**Authors:** Andrew R. Zullo, Melissa R. Riester, Kaleen N. Hayes, Yuan Zhang, Sarah D. Berry, Emmanuelle Belanger, Meghan A. Cupp, Francesca L. Beaudoin

**Affiliations:** 1 Department of Epidemiology, Brown University School of Public Health, 121 South Main Street, Box G-S121-2, Providence, RI 02912 USA; 2 Department of Health Services, Policy, and Practice, Brown University School of Public Health, Providence, RI USA; 3 Center for Gerontology and Healthcare Research, Brown University School of Public Health, Providence, RI USA; 4 Center of Innovation in Long-Term Services and Supports, Providence Veterans Affairs Medical Center, Providence, RI USA; 5 Graduate Department of Pharmaceutical Sciences, Faculty of Pharmacy, University of Toronto Leslie Dan, Toronto, ON Canada; 6 Hinda and Arthur Marcus Institute for Aging Research, Hebrew SeniorLife, Roslindale, MA USA; 7 Department of Medicine, Beth Israel Deaconess Medical Center, Boston, MA USA; 8 Harvard Medical School, Boston, MA USA

**Keywords:** Anti-Inflammatory Agents, Non-Steroidal, Electronic Health Records, Opioid, Rehabilitation

## Abstract

**Background:**

Although a majority of patients in the U.S. receive post-acute care in skilled nursing facilities (SNFs) following hip fracture, large-sample observational studies of analgesic prescribing and use in SNFs have not been possible due to limitations in available data sources. We conducted a proof-of-concept federated analysis of electronic health records (EHRs) from 11 SNF chains to describe analgesic use during hip fracture post-acute care.

**Methods:**

We included residents with a diagnosis of hip fracture between January 1, 2018 and June 30, 2021 who had at least one administration of an analgesic. Use of analgesics was ascertained from EHR medication orders and medication administration records. We quantified the proportion of residents receiving analgesic regimens based on the medications that were administered up to 100 days after hip fracture diagnosis. Plots visualizing trends in analgesic use were stratified by multiple resident characteristics including age and Alzheimer’s Disease and Related Dementias (ADRD) diagnosis.

**Results:**

The study included 23,706 residents (mean age 80.5 years, 68.6% female, 87.7% White). Most (~ 60%) residents received opioids + APAP. Monotherapy with APAP or opioids was also common. The most prevalent regimens were oxycodone + APAP (20.1%), hydrocodone + APAP (15.8%), APAP only (15.1%), tramadol + APAP (10.4%), and oxycodone only (4.3%). During the study period, use of APAP-only increased, opioids-only decreased, and opioids + APAP remained stable. Use of APAP-only appeared to be more prevalent among individuals aged > 75 years (versus ≤ 75 years) and those with ADRD (versus without).

**Conclusions:**

We successfully leveraged federated SNF EHR data to describe analgesic use among residents receiving hip fracture post-acute care.

**Supplementary Information:**

The online version contains supplementary material available at 10.1186/s12877-024-05486-0.

## Introduction

Pain management with analgesic medications is crucial during the post-acute care period following hip fracture hospitalization among older adults, as it can significantly impact functional recovery and prevent adverse outcomes such as delirium [1–5]. Pain management during post-acute hip fracture care is also important because undertreated pain may interfere with functional recovery through missed or shortened physical therapy sessions [1, 6–9]. The majority of individuals hospitalized for hip fracture are discharged to skilled nursing facilities (SNFs) for rehabilitation and pain management because hip fractures often result in severe pain and disability [10–17]. SNFs are therefore a critical place to understand and optimize analgesic prescribing practices following hip fracture.

Evidence-based guidance recommends multimodal analgesia, but does not specify which combinations of analgesics are safest and most effective for older adults receiving post-acute care in SNFs following hip fracture [8, 18–24]. Additionally, it is well known that the most common analgesics (e.g., opioids, non-steroidal anti-inflammatory drugs [NSAIDs]) have the potential for harms that may be potentiated by older age and polypharmacy. In order to effectively and safely treat pain in older adults with hip fracture, and to resolve equipoise around the best evidence for pain management, we must first understand which prescription (e.g., opioids) and non-prescription (e.g., acetaminophen [APAP], ibuprofen) analgesics are routinely administered during institutional post-acute SNF care.

The vast majority of patients in U.S. SNFs are insured by Medicare. However, large-sample observational studies using prescription drug claims data have not been possible because drug dispensing information is not captured by Medicare Part D claims during the SNF encounter. Payment for medications and other services during post-acute SNF care are bundled and covered by Medicare Part A. As a result, studies have: focused on analgesic prescribing during the hip fracture hospitalization rather than the post-acute care period; excluded individuals receiving short-term institutional post-acute care in SNFs, who comprise the majority of patients with hip fracture; or examined analgesic prescribing following discharge from institutional post-acute care [5, 25–43]. New data sources and approaches are necessary to overcome this barrier. Electronic health record (EHR) data from SNFs may be particularly well-suited to examine analgesic use in SNFs among older adults with hip fracture because information on medication prescribing and administrations are available, including for non-prescription medications. However, studies using SNF EHR data are scarce. A key reason for this scarcity is that each SNF or SNF chain’s customizations to their EHR system result in heterogeneous data that must harmonized into a single functional database.

In this proof-of-concept study, we explored the feasibility of harmonizing EHR data from 11 U.S. SNF chains into a single federated database to describe patterns of analgesic use in a large population of residents who were administered analgesics following hip fracture. In particular, we focused on describing administrations of the most common prescription and non-prescription analgesics administered in SNFs for post-fracture pain, including opioids, APAP, and NSAIDs.

## Methods

### Study design and data sources

In mid-2020, our institution partnered with several long-term care facility chains that use the PointClickCare® EHR system to conduct multiple studies related to the COVID-19 pandemic. Chains and facilities within those chains often customized their EHR systems, which introduced substantial heterogeneity in medication names, data structures, order types, and other data elements. Considerable additional heterogeneity was introduced by facility-specific practices related to the general operations of prescribing, including discontinuing and retiming medication orders and administrations. Thus, in this particular proof-of-concept study, we aimed to understand whether it was possible to harmonize medication order and administration data from multiple SNF chains into a single federated database.

Our observational study leveraged the EHR and Minimum Data Set (MDS) data from 11 of 12 possible chains, which comprised nearly 700 U.S. SNFs, between 2018 and 2021. Electronic health record data included information on the daily census in each facility, resident demographics, diagnosis codes, medication orders, and barcode medication administration records (MAR). The EHR also contained MDS data, which are scheduled government-mandated assessments that document clinical resident information at days 5, 14, 30, 60, and 90 after admission to the SNF for post-acute care. Additional unscheduled assessments are required and administered under specific circumstances.

This study was approved by the Brown University Institutional Review Board. Due to the use of deidentified administrative data, the need for informed consent was waived.

### Study population

We included SNF residents with a diagnosis of hip fracture documented on the EHR diagnosis sheet admission record in the principal position between January 1, 2018 and June 30, 2021 (Additional Table 1) and at least one administration of an analgesic medication in the MAR in the 100 days following the hip fracture diagnosis date (Additional Fig. 1). We excluded residents without an MDS assessment in the 33 days following hip fracture diagnosis to allow resident characteristics to be ascertained. We also excluded those with missing information on age, sex, cognitive function, or physical function. Residents were followed from the hip fracture diagnosis date, also referred to as baseline, for up to 100 days after the hip fracture diagnosis date. We chose to follow individuals for up to 100 days as this aligns with the maximum number of days for Medicare’s SNF care benefit coverage.

**Table 1:** Characteristics of patients admitted to U.S. skilled nursing facilities after hip fracture and receiving analgesic medications between January 1, 2018 and June 30, 2021 (*N* = 23,706)

Baseline Characteristics*	Overall	Prior to the Onset of COVID-19 Pandemic^†^	After the Onset of COVID-19 Pandemic^†^	SMD, Pre- vs Post-Pandemic Periods
N (%)	23,706 (100)	16,690 (29.6)	7,016 (70.4)	
Age at SNF Admission, mean (SD)	80.5 (10.8)	80.5 (10.8)	80.6 (10.5)	0.01
Female Sex	16,265 (68.6)	11,480 (68.8)	4,785 (68.2)	-0.01
Race/Ethnicity				
White	20,784 (87.7)	14,664 (87.9)	6,120 (87.2)	-0.02
Black or African American	932 (3.9)	642 (3.8)	290 (4.1)	0.01
Hispanic or Latino	511 (2.2)	357 (2.1)	154 (2.2)	0.00
Other race	749 (3.2)	523 (3.1)	226 (3.2)	0.00
Missing	730 (3.1)	504 (3.0)	226 (3.2)	0.01
ADL Score, mean (SD)^‡^	17.7 (3.3)	17.7 (3.2)	17.8 (3.5)	0.02
Independent to limited assistance required	3,174 (13.4)	2,182 (13.1)	992 (14.1)	0.03
Extensive assistance required	15,007 (63.3)	10,976 (65.8)	4,031 (57.5)	-0.17
Extensive dependency	5,525 (23.3)	3,532 (21.2)	1,993 (28.4)	0.17
Cognitive Function^§^				
Intact to mild impairment	18,540 (78.2)	13,138 (78.7)	5,402 (77.0)	-0.04
Moderate impairment	4,299 (18.1)	2,949 (17.7)	1,350 (19.2)	0.04
Severe impairment	867 (3.7)	603 (3.6)	264 (3.8)	0.01
Pain				
No pain	3,720 (15.7)	2,528 (15.1)	1,192 (17.0)	0.05
Mild/infrequent pain	10,728 (45.3)	7,721 (46.3)	3,007 (42.9)	-0.07
Severe/frequent pain	6,773 (28.6)	4,808 (28.8)	1,965 (28.0)	-0.02
Missing	2,485 (10.5)	1,633 (9.8)	852 (12.1)	0.08
Active Medical Conditions^||^				
ADRD	5,983 (25.2)	4,138 (24.8)	1,845 (26.3)	0.03
Arthritis	5,296 (22.3)	3,643 (21.8)	1,653 (23.6)	0.04
Atrial fibrillation	5,844 (24.7)	4,041 (24.2)	1,803 (25.7)	0.03
Chronic pulmonary disease	4,989 (21.0)	3,383 (20.3)	1,606 (22.9)	0.06
Coronary artery disease	4,623 (19.5)	3,162 (18.9)	1,461 (20.8)	0.05
Diabetes	5,912 (24.9)	4,082 (24.5)	1,830 (26.1)	0.04
GERD or ulcer	6,684 (28.2)	4,606 (27.6)	2,078 (29.6)	0.04
Heart failure	3,683 (15.5)	2,552 (15.3)	1,131 (16.1)	0.02
Renal disease	4,607 (19.4)	3,014 (18.1)	1,593 (22.7)	0.12
Stroke, TIA, or CVA	1,166 (4.9)	672 (4.0)	494 (7.0)	0.13
Depression	7,256 (30.6)	5,019 (30.1)	2,237 (31.9)	0.04
Cancer	2,012 (8.5)	1,427 (8.6)	585 (8.3)	-0.01
Number of Active Medical Conditions, mean (SD)^||^	6.4 (3.1)	6.2 (3.1)	6.8 (3.2)	0.19
Gagne Combined Comorbidity Index, mean (SD)^**^	3.4 (2.2)	3.3 (2.2)	3.7 (2.3)	0.17

*Abbreviations: SNF* Skilled nursing facility, *SMD* Standardized mean difference, *SD* Standard deviation, *CFS* Cognitive Function Scale, *ADRD* Alzheimer’s disease and related dementias, *GERD* Gastroesophageal reflux disease, *TIA* Transient ischemic attack, *CVA* Cerebrovascular accident, *MDS* Minimum Data Set, *ICD-10* International Classification of Diseases version 10

^*^At or on the MDS assessment recorded closest to the time of admission to the *SNF*

^†^Onset of COVID 19 Pandemic Considered as March 16, 2020 or later. Pre-pandemic period includes residents with a hip fracture diagnosis date between January 1, 2018 and March 15, 2020. The post-pandemic period is from March 16, 2020 to June 30, 2021

^‡^Measured using the Minimum Data Set Morris 28-point scale of Independence in Activities of Daily Living and categorized as: 0 to 14 (independent to limited assistance required), 15 to 19 (extensive assistance required), 20 or higher (extensive dependency)

^§^Measured using Minimum Data Set Cognitive Function Scale, a 4-point scale of cognitive function categorized as: 1–2 (intact to mild impairment), 3 (moderate impairment), and 4 (severe impairment)

^||^Ascertained using the MDS Section I Active Diagnoses, which includes 56 conditions

^**^Ascertained using ICD-10 codes from the diagnosis sheet documentation in the electronic health records; ranges from -2 to 26

**Fig. 1:**
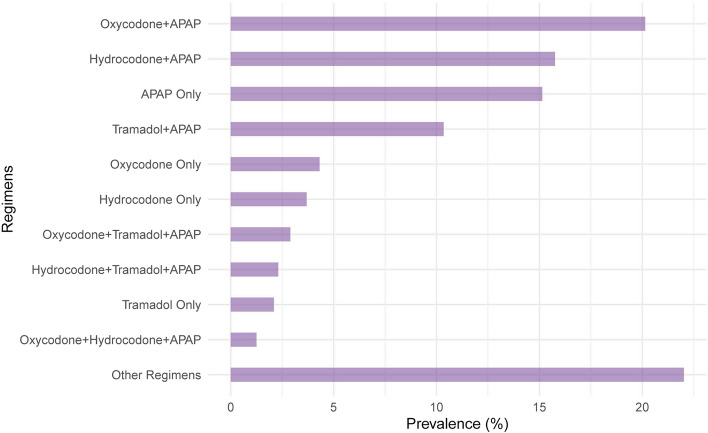
The most common analgesic medication regimens used among patients in U.S. skilled nursing facilities after hip fracture between January 1, 2018 and June 30, 2021 (*N* = 23,706). Presents proportion of residents who had at least one administration of the medication(s) in a given regimen at any point between the hip fracture diagnosis and 100 days following the hip fracture diagnosis. Analgesic regimens are mutually exclusive categories. Use of all other individual medications/medication combinations (e.g., ibuprofen, ibuprofen + APAP) are represented in the “Other Regimens” category. Abbreviations: APAP, acetaminophen

### Analgesic medications

Medication use was ascertained by linking EHR medication orders to barcode MARs for prescription and non-prescription medications that were ordered during a resident’s entire SNF encounter. The orders data provided information such as medication name, medication strength, start date, and discontinued date. The MAR data included elements such as medication directions, the exact date and time of each medication administration, and dose administered. We identified use of analgesics by restricting to standing and pro-re-nata (“as needed”) orders for APAP, opioids, and NSAIDs that were administered at least once in the MAR between the hip fracture diagnosis date and 100 days after the hip fracture diagnosis date (Additional Table 2). Given that this was a proof-of-concept study, we chose to examine a limited number of analgesic classes that we expected a priori to be used most frequently to manage pain following a hip fracture [8, 18–24].

### Resident characteristics

Baseline demographic information (age, sex, race/ethnicity) and clinical characteristics were ascertained from the SNF admission MDS assessment or the first available MDS assessment, which was typically completed shortly after SNF admission but could occur up to 33 days after the hip fracture date on the SNF diagnosis sheet admission record. All characteristics were therefore ascertained after the hip fracture hospitalization in the post-fracture period rather than before the hip fracture occurred. Clinical characteristics included active conditions, functional status based on the Morris 28-point scale of independence in activities of daily living [44], cognitive function based on the Cognitive Function Scale [45], and pain level based on the Centers for Medicare and Medicaid Services quality indicator definition (no pain, mild/infrequent pain, severe/frequent pain) [46]. We measured multimorbidity two ways: 1) based on the number of active conditions listed in the MDS (56 total active conditions) and 2) using the Gagne Combined Comorbidity Score [47], which was modified to include International Classification of Diseases, tenth revision, diagnosis codes documented in the EHR and active conditions in the MDS.

### Statistical analyses

We described the use of analgesic regimens during the SNF episode two ways: based on 1) individual analgesic medications (e.g., oxycodone + APAP) and 2) analgesic classes (e.g., opioids + APAP). Because of the multitude of different regimens/complexity of measuring regimen changes, we chose to classify patients based on the combination of medications used at any time during the SNF stay and during calendar quarters (i.e., a person classified as having a regimen of oxycodone + APAP may have been administered both medications simultaneously or may have been administered monotherapy with opioids for a period of time followed by monotherapy with APAP).

We quantified the frequency and percentage of residents in the study population who received the top 25 combinations of individual analgesic medications. Regimens were categorized into mutually exclusive groups based on the individual analgesic medication(s) that were administered at any point during follow-up. Given that the onset of the COVID-19 pandemic occurred during the study period, we conducted exploratory analyses to compare resident characteristics and patterns of analgesic administrations before (hip fracture diagnosis date between January 1, 2018 to March 15, 2020) versus after (March 16, 2020 to June 30, 2021) the onset of the pandemic. Resident characteristics were compared using standardized mean differences (SMDs). We also reported changes in the use of combinations of individual analgesic medications across time periods by calculating unadjusted risk ratios (RR) and risk differences (i.e., percentage point differences [PPD]) with 95% confidence intervals (95%CI) using modified Poisson regression and linear regression models with robust standard errors.

In addition, we plotted the use of analgesic regimens in each calendar quarter of our study period to visualize trends in analgesic medication classes over time. In each calendar quarter, use of an analgesic regimen was calculated as the number of residents who were administered at least one dose of the medication(s) in that regimen at any point in that calendar quarter divided by the number of residents who were present in the SNFs and received at least one dose of any analgesic in that calendar quarter. Residents with no analgesic administrations in a given quarter would not be represented in that quarter, even if they had an active order for analgesics. Given the potential for heterogeneity in trends across resident subgroups, we also stratified plots by key characteristics that might be expected to influence analgesic regimen receipt, including age, sex, race, severity of cognitive impairment, Alzheimer’s Disease and Related Dementias (ADRD) diagnosis, physical impairment, pain severity, and comorbidity burden. Plots indicated the start of the COVID-19 pandemic to visualize time trends in the use of analgesic regimens in the pre- and post-pandemic periods.

### Software

Analyses were performed using SAS, version 9.4 (SAS Institute, Cary, NC, United States) and R software, version 4.1.3 (R Foundation for Statistical Computing, Vienna, Austria).

## Results

### Study population

The final study population included 23,706 SNF residents with hip fracture and analgesic use (Additional Fig. 1). The mean (standard deviation [SD]) age was 80.5 (10.8) years, 16,265 (68.6%) were female, and 20,784 (87.7%) were White (Table 1). A majority of residents required extensive assistance in their activities of daily living (63.3%), 18.1% had moderate cognitive impairment, and the mean (SD) Gagne combined comorbidity score was 3.4 (2.2). Most residents had mild/infrequent pain (45.3%), 28.6% had severe/frequent pain, and 15.7% had no pain based on the first completed pain assessment.

Residents after the onset of the pandemic had greater extensive dependency in activities of daily living (28.4% versus 21.2%, SMD 0.17), multimorbidity (mean [SD] Gagne combined comorbidity score 3.7 [2.3] vs. 3.3 [2.2], SMD 0.17), and a greater proportion had renal disease (22.7% vs. 18.1%, SMD 0.12), and stroke, transient ischemic attack, or cerebrovascular accident (7.0% vs. 4.0%, SMD 0.13) (Table 1).

### Individual analgesic medications administered after hip fracture

Overall, the most common analgesic medications during the SNF stay after hip fracture were oxycodone + APAP (20.1%), hydrocodone + APAP (15.8%), APAP only (15.1%), and tramadol + APAP (10.4%) (Fig. 1; Additional Table 3). Other moderately prevalent regimens included oxycodone only (4.3%), hydrocodone only (3.7%), oxycodone + tramadol + APAP (2.9%), hydrocodone + tramadol + APAP (2.3%), and tramadol only (2.1%).

When comparing the prevalence of analgesic use in the period before versus after the onset of the COVID-19 pandemic, the proportion of individuals with oxycodone + APAP increased (RR = 1.12, 95%CI 1.06 to 1.18; PPD = 2.28, 95%CI 1.15 to 3.42) and APAP only increased (RR = 1.13, 95%CI 1.06 to 1.21; PPD = 1.90, 95%CI 0.88 to 2.92). Use of hydrocodone + APAP decreased (RR = 0.91, 95%CI 0.85 to 0.98; PPD = -1.42, 95%CI -2.42 to -0.42) as did use of hydrocodone only (RR = 0.63, 95%CI 0.54 to 0.74; PPD = -1.54, 95%CI -2.02 to -1.06) after the onset of the COVID-19 pandemic.

### Analgesic medication classes administered after hip fracture

**Fig. 2:**
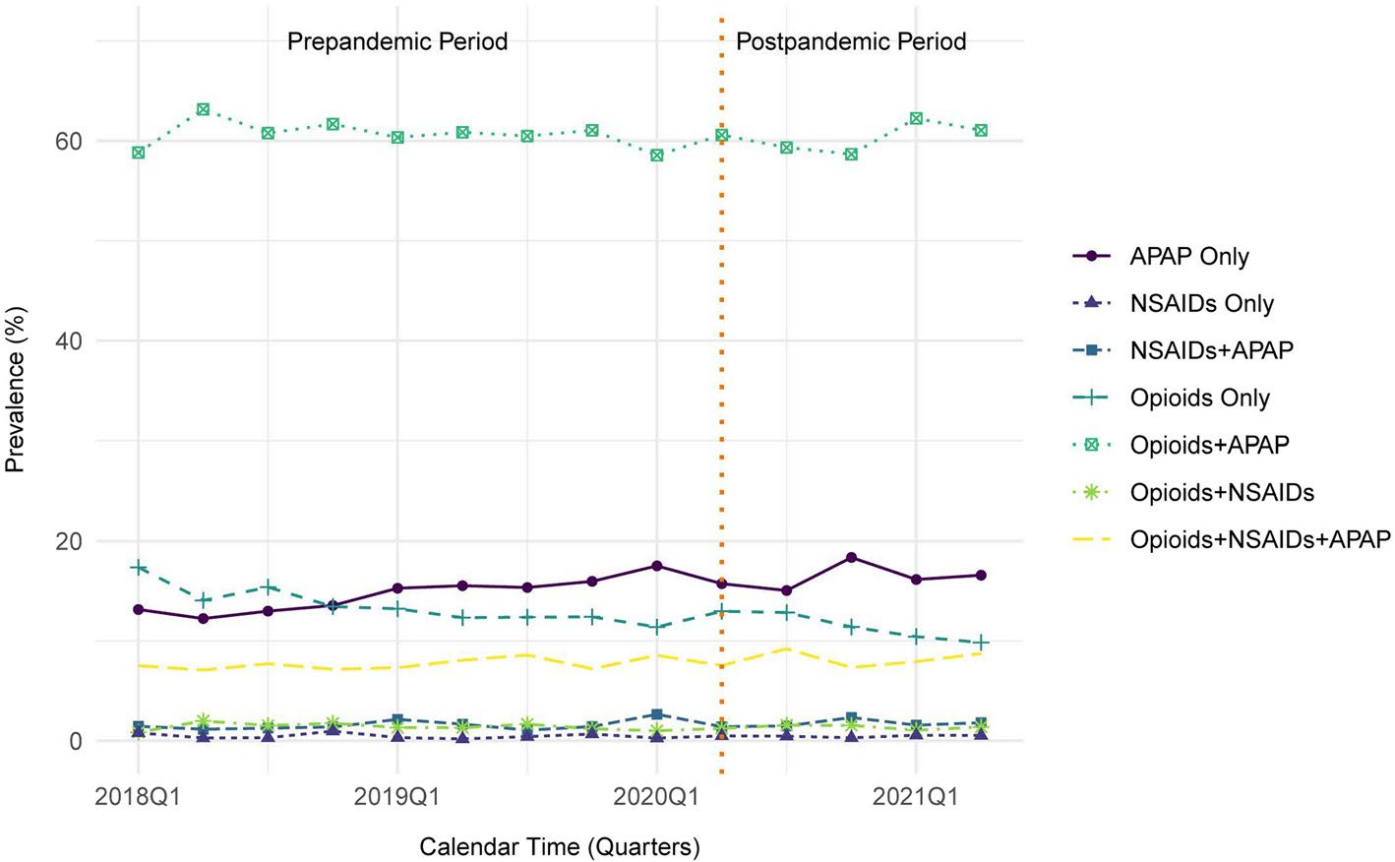
Trends in the use of analgesic medication class-level regimens among patients in U.S. skilled nursing facilities after hip fracture between January 1, 2018 and June 30, 2021. Presents proportion of residents who had at least one administration of the medication class(es) in a given regimen at any point during the quarter of calendar time. Analgesic medication class-level regimens are mutually exclusive categories. The denominator used to calculate the proportion in each quarter is the number of residents who were present in the skilled nursing facilities and received at least one dose of any analgesic in that quarter. Abbreviations: APAP, acetaminophen; NSAIDs, non-steroidal anti-inflammatory drugs

Between January 1, 2018 and June 30, 2021, APAP only regimens increased, opioid only regimens decreased, and both opioids + APAP and opioids + NSAIDs + APAP regimens remained stable over time (Fig. 2).

**Fig. 3:**
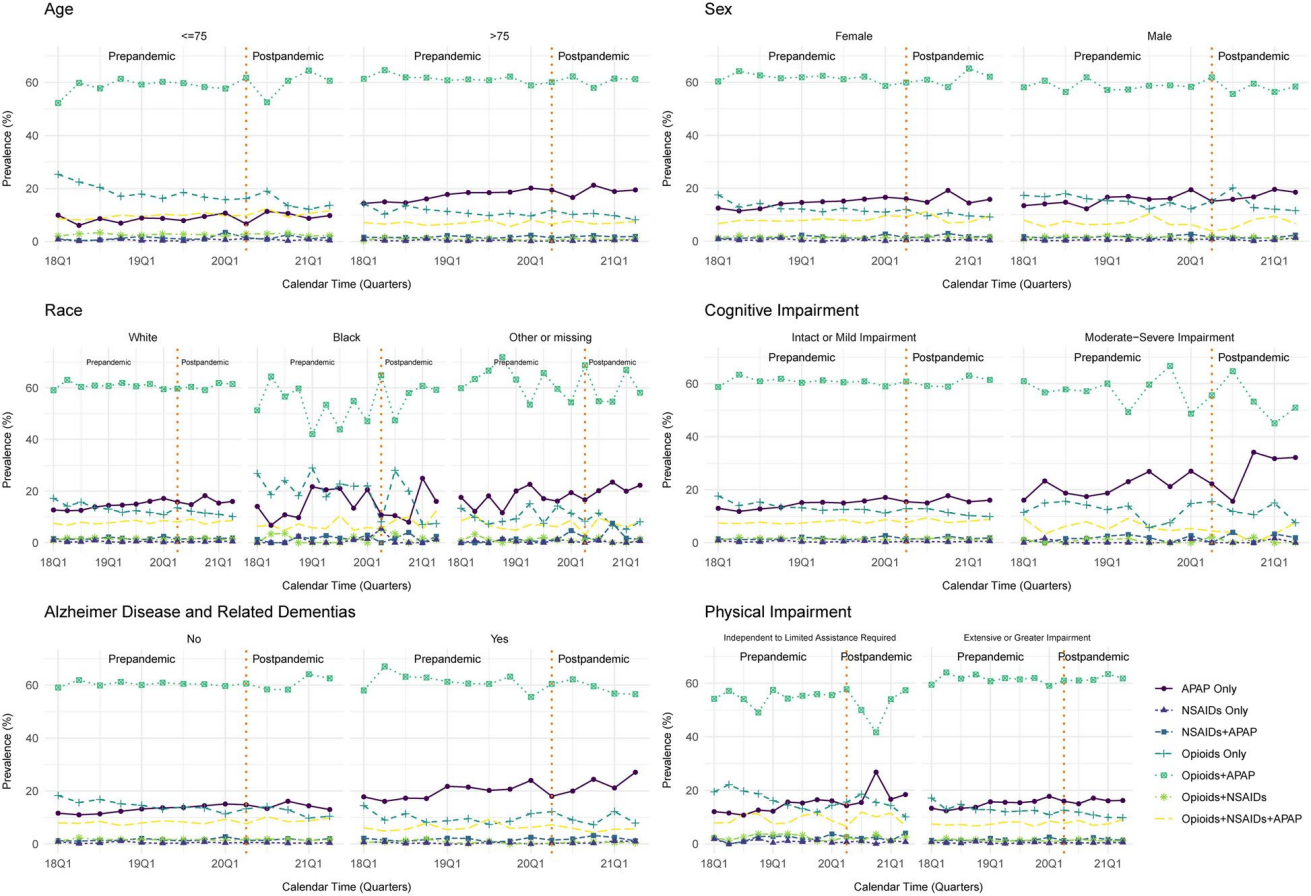
Trends in the use of analgesic medication class-level regimens among patients in U.S. skilled nursing facilities after hip fracture between January 1, 2018 and June 30, 2021 stratified by key patient subgroups. Presents proportion of residents who had at least one administration of the medication class(es) in a given regimen at any point during the quarter of calendar time among key subgroups. Analgesic medication class-level regimens are mutually exclusive categories. The denominator used to calculate the proportion in each quarter is the number of residents who were present in the skilled nursing facilities and received at least one dose of any analgesic in that quarter. Abbreviations: APAP, acetaminophen; NSAIDs, non-steroidal anti-inflammatory drugs

**Fig. 4:**
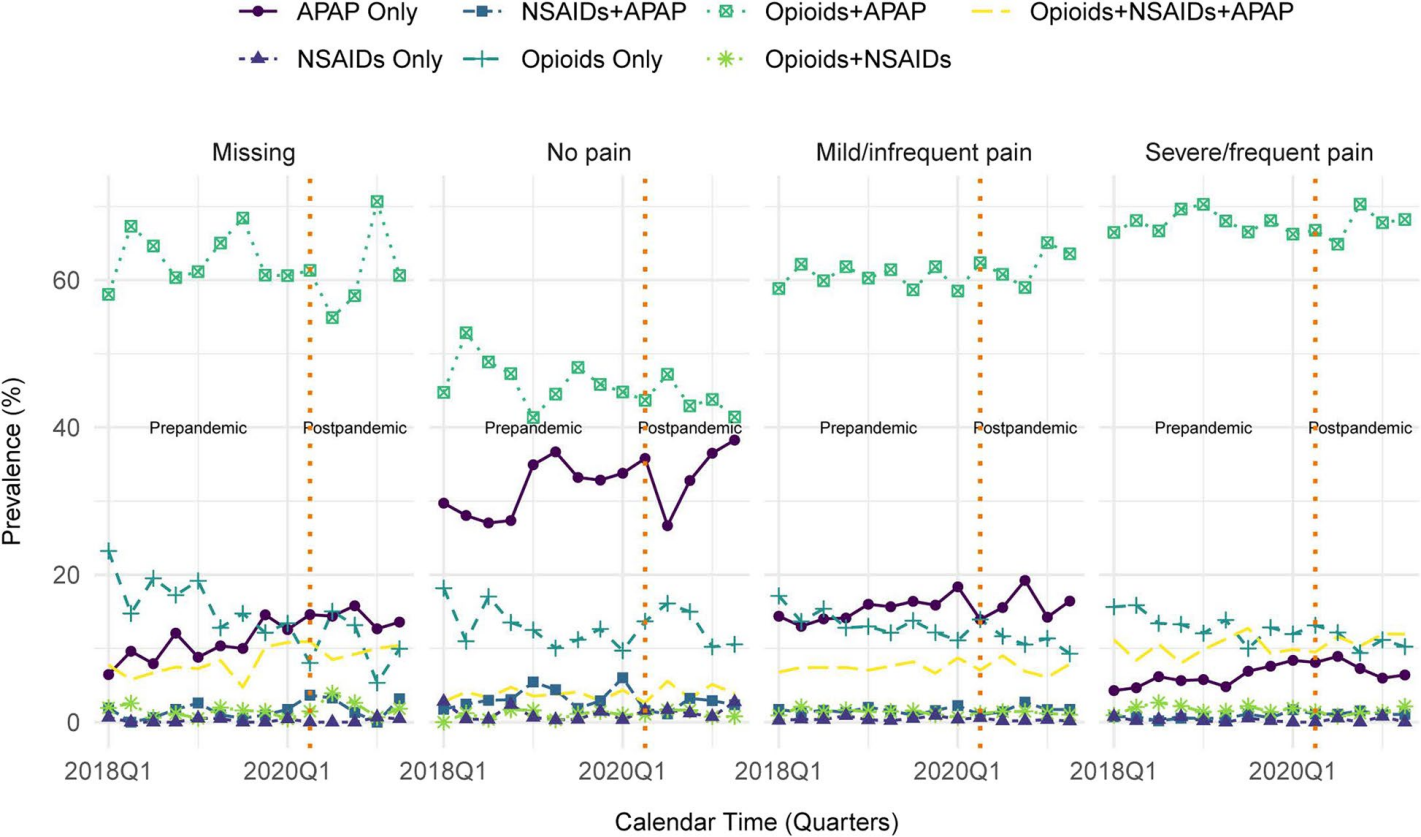
Trends in the use of analgesic medication class-level regimens among patients in U.S. skilled nursing facilities after hip fracture between January 1, 2018 and June 30, 2021 stratified by pain severity. Presents proportion of residents who had at least one administration of the medication class(es) in a given regimen at any point during the quarter of calendar time among patients in each pain severity subgroup. Analgesic medication class-level regimens are mutually exclusive categories. The denominator used to calculate the proportion in each quarter is the number of residents who were present in the skilled nursing facilities and received at least one dose of any analgesic in that quarter. Abbreviations: APAP, acetaminophen; NSAIDs, non-steroidal anti-inflammatory drugs

Trends were generally similar when stratifying on age, sex, race, cognitive impairment, ADRD, physical impairment, or multimorbidity (Fig. 3; Additional Fig. 2). Use of APAP only appeared to be more prevalent and increasing among individuals aged older than 75 years (versus 75 years or younger) and those with ADRD (versus those without). When stratifying by pain quality indicator, opioid + APAP regimens were most prevalent among individuals with severe/frequent pain and least prevalent among those with no pain, but there appeared to be no remarkable or differential time trends by pain severity/frequency (Fig. 4).

## Discussion

In this proof-of-concept study, we successfully harmonized and then leveraged EHR medication order and administration data to describe analgesic medications that were administered to SNF residents following a hip fracture. We found that a majority (~ 60%) of residents in 11 U.S. SNF chains received opioids + APAP in a SNF in the 100 days after hip fracture. Oxycodone + APAP, hydrocodone + APAP, and tramadol + APAP were some of the most prevalent regimens, each with a prevalence greater than 10%. Acetaminophen only was also among the top analgesic regimens (15.1% of residents). Overall, we found that SNF EHR data is a rich source of information on medication prescribing and administrations for individuals receiving institutional hip fracture post-acute care, and provides information that is not captured by other commonly used datasets (e.g., Medicare Part D claims). Future research should consider leveraging SNF EHR data to examine the safety, effectiveness, and appropriateness of analgesic prescribing and administrations during post-acute care in SNFs following hip fracture.

Although a majority of individuals hospitalized for hip fracture are discharged to SNFs for post-acute care, limitations in most data sources prevent the ascertainment of information on medication prescribing and administrations during institutional post-acute care. Thus, limited evidence currently exists on analgesic use in SNFs during the post-acute care period. This study overcame those limitations because we leveraged EHR medication orders data and barcode MARs to identify both analgesic orders and administrations during the SNF stay. We also described the use of non-prescription analgesics, like ibuprofen, which are not reliably captured in insurance claims data.

Multimodal analgesia is often recommended to manage acute pain following hip fracture [8, 18–23]. However, the safest and most effective oral analgesic regimens are unknown for older adults receiving post-acute care in SNFs. Individuals receiving SNF care may be especially vulnerable to the adverse effects of opioids (e.g., sedation, falls) and NSAIDs (e.g., impaired renal function, bleeding) because many residents are older, multimorbid, and have polypharmacy. Our results suggest that opioids + APAP are the most common regimen among SNF residents after hip fracture, followed by monotherapy with APAP or opioids. Use of NSAIDs, alone or in combination, was infrequent in our data. Thus, it may be appropriate for future studies examining the safety and effectiveness of analgesics during post-acute care for hip fracture to focus on non-NSAID regimens by comparing opioids + APAP, APAP only, and opioids only. A study comparing the safety and effectiveness of oxycodone + APAP, hydrocodone + APAP, and tramadol + APAP would also be beneficial because the 2023 American Geriatrics Society Beers Criteria list tramadol as a drug to be used with caution in older adults. Additional descriptive research is needed to inform safety and effectiveness studies that aim to compare more specific analgesic regimens (i.e., that include analgesic doses, scheduling [pro-re-nata or scheduled administrations], frequency of administrations, and sequences of analgesics [e.g., oxycodone + APAP versus oxycodone only followed by APAP only]). It may be beneficial for these future studies to link EHR data to other sources (e.g., MDS, Medicare claims) to ascertain information on residents’ clinical status, medication use, and conditions that may cause pain prior to and during the SNF stay.

We found that the use of an analgesic regimen containing APAP only appeared to be more prevalent and increasing over time among residents with ADRD, but was stable for residents without ADRD. This increasing trend in APAP only use for residents with ADRD was occurring prior to the onset of the COVID-19 pandemic and continued to increase afterwards. Data suggest that individuals with cognitive impairment are less likely to receive opioids, receive lower doses of opioids, and are less likely to report pain during hospitalization for hip fracture [30, 31, 34, 39]. Evaluating the use of pro-re-nata analgesic medications for SNF residents with and without cognitive impairment is a particularly important area of future research, since individuals with cognitive impairment who cannot communicate their pain level are at risk of undertreatment of pain because they must rely on an observant staff or family member to identify their pain and request that analgesics are administered.

Results from our study are some of the first to examine how trends in analgesic prescribing in SNFs for residents with hip fracture may have been impacted by the COVID-19 pandemic. Although the use of some individual medications/medication combinations differed after the onset of the pandemic, it was reassuring to see that the use of opioids + APAP remained consistent throughout the study period because opioid therapy plays an important role in the management of acute pain related to traumatic injuries and moderate to severe postoperative pain [48]. Notably, the onset of the pandemic probably did not have a homogenous effect on facilities across the U.S., where outbreaks occurred in different regions of the country at varying times. Further examination may be useful to understand the association between increasing prevalence of COVID-19 in a geographic region or outbreaks within the facility and changes to analgesic prescribing and administrations. Such information is useful to understand facility characteristics that are associated with higher or lower quality pain management practices for residents following hip fracture.

### Limitations

Our study has several potential limitations, which also represent important lines of future research. First, our results may not generalize well to residents outside of the 11 SNF chains providing our EHR data. Given that the diagnosis of hip fracture was based on the EHR diagnosis sheet (rather than inpatient hospitalization claims), we identified both residents who were admitted to the SNF following a hip fracture hospitalization and, potentially, some individuals who experienced a hip fracture during the SNF stay. Future research efforts should involve even larger-scale EHR data that can be linked to other data sources (i.e., hospitalization claims). Such data are increasingly available from the newly established Long-Term Care Data Cooperative, which is the descendant of the effort we report here [49].

Second, in our data, linking the EHR medication orders to MARs restricted to orders that were administered at least once, thus excluding individuals who were not administered at least one dose of analgesics in the 100 days following hip fracture. Nearly all individuals receiving SNF care after hip fracture will receive analgesic medications. As shown in Additional Fig. 1, approximately 96% of individuals with hip fracture had an analgesic medication administration at some time. For that reason, we focused our inferences on individuals who received at least one analgesic administration. Important questions remain to be answered about the roughly 4% of individuals who do not appear to receive any analgesic medications.

Third, the EHR data could not be linked to insurance claims data (e.g., Medicare) due to data use agreements between our institution and the SNF chains. Thus, inpatient hospitalization claims, claims for outpatient office visits, and prescription drug dispensings prior to the SNF stay were unavailable, limiting our ability to ascertain information on clinical characteristics and medication use prior to the SNF stay. In particular, we were unable to measure surgical procedures performed during the hospitalization prior to SNF admission. Similarly, we were not permitted to link the EHR data to datasets that provide information on SNF characteristics, such as the Certification and Survey Provider Enhanced Reporting (CASPER) system data. We were therefore unable to describe the characteristics of the SNFs (e.g., staffing, profit status).

Fourth, this proof-of-concept study focused on a limited number of common oral analgesic regimens. It did not include all multimodal treatments that could be used to alleviate pain post-fracture. Pharmacologic approaches that are not widely recommended by guidelines and that have little evidence to support their use for hip fracture pain, such as gabapentin or lidocaine patches, were not examined. Non-pharmacological approaches (e.g., hot and cold packs) were also not investigated. Future work to examine whether gabapentin and other treatments are being used as opioid-sparing treatment strategies in SNFs after injuries and surgical procedures might be a particularly important area of future research. Understanding medication switching and dosing strategies (i.e., “trajectories”) throughout the post-acute care stay, including opioid de-intensification and tapering, is also a high priority area of future research that should leverage a federated database of SNF EHRs.

Finally, our EHR data are generated in the course of usual care and have not yet been extensively validated. Future studies should compare EHR data to other datasets to investigate whether there is concordance in measures that are common between datasets. While the federation of data from multiple SNF chains was ultimately successful, we encountered several challenges that necessitate additional validation work to ensure high accuracy, including variation in data structures and medication documentation practices across chains and SNFs. These differences required a substantial initial effort to harmonize the data and ensure consistency. Additionally, integrating MARs with prescription order data presented technical difficulties, as MARs were managed differently across SNF chains. For example, medications could be discontinued, modified, or retimed without clear documentation. Overcoming these challenges provided valuable insights into the process of conducting federated analyses with EHR data and highlighted areas for improvement in future studies.

## Conclusions

In conclusion, SNF EHR data is a rich source of information on medication prescribing and administrations, which can be leveraged to study prescription and non-prescription analgesic use during institutional post-acute care for many conditions. A majority of SNF residents in our study population received opioids + APAP to manage pain in the 100 days following hip fracture, while analgesic regimens with APAP or opioid monotherapy were moderately prevalent. Our results suggest that it is feasible for a future study to compare the benefits and harms of opioids + APAP, APAP only, and opioids only among SNF residents post-hip fracture. A better understanding of the safety and effectiveness of analgesic regimens during post-acute care for hip fracture in SNFs is critical because residents are vulnerable to medication adverse effects, yet concerns about the safety of these drugs may manifest in the undertreatment of pain, which can impair rehabilitation and functional recovery.

## Supplementary Information

Supplementary Material: Additional file 1. Identification of hip fracture diagnoses. Additional Table 2. Analgesic medications. Additional Table 3. Analgesic medication regimens administered to patients admitted to U.S. skilled nursing facilities after hip fracture between January 1, 2018 and June 30, 2021 (*N*=23,706). Additional Figure 1. Flow diagram of the study population. Additional Figure 2. Trends in the use of analgesic medication class-level regimens among patients in U.S. skilled nursing facilities after hip fracture between January 1, 2018 and June 30, 2021 stratified by subgroups of the Gagne Combined Comorbidity Score.

## Data Availability

The data that support the findings were obtained through a partnership between Brown University and 11 U.S. nursing home chains. Restrictions apply to the availability of these data, which were used under data use agreements with each chain for the current study, and so are not publicly available. However, similar data can now be accessed through the Long-Term Care Data Cooperative, which provides a standard application process that researchers can use to access the data.
