# Supplementary material for: Analgesic regimens administered to older adults receiving skilled nursing facility care following hip fracture: a proof-of-concept federated analysis
Source: BMC Geriatr. 2024 Oct 30;24:897. doi: 10.1186/s12877-024-05486-0 (PMC11523817; doi:10.1186/s12877-024-05486-0)
Supplement: Supplementary file 1 — Supplementary Material: Additional file 1. Identification of hip fracture diagnoses. Additional Table 2. Analgesic medications. Additional Table 3. Analgesic medication regimens administered to patients admitted to U.S. skilled nursing facilities after hip fracture between January 1, 2018 and June 30, 2021 (N=23,706). Additional Figure 1. Flow diagram of the study population. Additional Figure 2. Trends in the use of analgesic medication class-level regimens among patients in U.S. skilled nursing facilities after hip fracture between January 1, 2018 and June 30, 2021 stratified by subgroups of the Gagne Combined Comorbidity Score. [file 12877_2024_5486_MOESM1_ESM.docx]

**SUPPLEMENTARY MATERIALS**

**Title:** Analgesic Regimens Administered to Older Adults Receiving Skilled Nursing Facility Care Following Hip Fracture: A Proof-of-Concept Federated Analysis

**Additional Table 1.** Identification of hip fracture diagnoses.

**Additional Table 2.** Analgesic medications.

**Additional Table 3.** Analgesic medication regimens administered to patients admitted to U.S. skilled nursing facilities after hip fracture between January 1, 2018 and June 30, 2021 (N=23,706).

**Additional Figure 1.** Flow diagram of the study population.

**Additional Figure 2.** Trends in the use of analgesic medication class-level regimens among patients in U.S. skilled nursing facilities after hip fracture between January 1, 2018 and June 30, 2021 stratified by subgroups of the Gagne Combined Comorbidity Score.

**Additional Table 1.** Identification of hip fracture diagnoses.

| **Hip Fracture ICD-10 Codes** | **Data Source** | **Coding Position** |
| --- | --- | --- |
| Starts with:  S72.0  S72.1  M80.05  M84.45  M80.85  M84.65 | Electronic health record “diagnosis sheet”: Skilled nursing facility admission record containing principal diagnosis, admitting diagnosis, and up to 24 additional secondary diagnoses | Principal position |
| Abbreviations: ICD-10, International Classification of Diseases, Tenth Revision. | | |

**Additional Table 2.** Analgesic medications.

| **Medication Class** | **Medications** |
| --- | --- |
| Acetaminophen | Acetaminophen |
| Opioids | Hydrocodone, morphine, oxycodone, tramadol, buprenorphine, butorphanol, codeine, fentanyl, hydromorphone, levorphanol, methadone, meperidine, oxymorphone, remifentanil, sufentanil, tapentadol |
| NSAIDs | Ibuprofen, naproxen, diclofenac, nabumetone, etodolac, celecoxib |
| Abbreviations: NSAIDs, non-steroidal anti-inflammatory drugs. | |

**Additional Table 3.** Analgesic medication regimens administered to patients admitted to U.S. skilled nursing facilities after hip fracture between January 1, 2018 and June 30, 2021 (N=23,706).

| **Analgesic Medication Regimen** | **Overall Use, N=23,706** | **Use Prior to the Onset of COVID-19 Pandemic^1^, N=16,690** | **Use After the Onset of COVID-19 Pandemic^1^, N=7,016** | **Risk Ratio^2^ (95% CLs), p-value** | **Percentage Point Difference^2^** **(95% CLs), p-value** |
| --- | --- | --- | --- | --- | --- |
| Oxycodone+APAP | 4,775 (20.1) | 3,249 (19.5) | 1,526 (21.8) | 1.12 (1.06, 1.18), p<0.01 | 2.28 (1.15, 3.42), p<0.01 |
| Hydrocodone+APAP | 3,737 (15.8) | 2,701 (16.2) | 1,036 (14.8) | 0.91 (0.85, 0.98), p<0.01 | -1.42 (-2.42, -0.42), p<0.01 |
| APAP Only | 3,589 (15.1) | 2,433 (14.6) | 1,156 (16.5) | 1.13 (1.06 1.21), p<0.01 | 1.90 (0.88, 2.92), p<0.01 |
| Tramadol+APAP | 2,454 (10.4) | 1,713 (10.3) | 741 (10.6) | 1.03 (0.95, 1.12), p=0.49 | 0.30 (-0.56, 1.15), p=0.49 |
| Oxycodone Only | 1,025 (4.3) | 726 (4.4) | 299 (4.3) | 0.98 (0.86, 1.12), p=0.76 | -0.09 (-0.65, 0.48), p=0.76 |
| Hydrocodone Only | 875 (3.7) | 692 (4.2) | 183 (2.6) | 0.63 (0.54, 0.74), p<0.01 | -1.54 (-2.02, -1.06), p<0.01 |
| Oxycodone+Tramadol+APAP | 687 (2.9) | 496 (3.0) | 191 (2.7) | 0.92 (0.78, 1.08), p=0.30 | -0.25 (-0.71, 0.21), p=0.29 |
| Hydrocodone+Tramadol+APAP | 547 (2.3) | 426 (2.6) | 121 (1.7) | 0.68 (0.55, 0.83), p<0.01 | -0.83 (-1.22, -0.44), p<0.01 |
| Tramadol Only | 499 (2.1) | 347 (2.1) | 152 (2.2) | 1.04 (0.86, 1.26), p=0.67 | 0.09 (-0.32, 0.49), p=0.67 |
| Oxycodone+Hydrocodone+APAP | 297 (1.3) | 205 (1.2) | 92 (1.3) | 1.07 (0.84, 1.36), p=0.60 | 0.08 (-0.23, 0.40), p=0.60 |
| Morphine+APAP | 190 (0.8) | 128 (0.8) | 62 (0.9) | 1.15 (0.85, 1.56), p=0.36 | 0.12 (-0.14, 0.37), p=0.37 |
| Oxycodone+Morphine+APAP | 170 (0.7) | 115 (0.7) | 55 (0.8) | 1.14 (0.83, 1.57), p=0.43 | 0.10 (-0.15, 0.34), p=0.44 |
| Oxycodone+APAP+Diclofenac | 169 (0.7) | 96 (0.6) | 73 (1.0) | 1.81 (1.34, 2.45), p<0.01 | 0.47 (0.20, 0.73), p<0.01 |
| Hydromorphone+APAP | 150 (0.6) | 114 (0.7) | 36 (0.5) | 0.75 (0.52, 1.09), p=0.13 | -0.17 (-0.38, 0.04), p=0.11 |
| Oxycodone+Ibuprofen+APAP | 143 (0.6) | 93 (0.6) | 50 (0.7) | 1.28 (0.91, 1.80), p=0.16 | 0.16 (-0.07, 0.38), p=0.18 |
| Ibuprofen+APAP | 142 (0.6) | 96 (0.6) | 46 (0.7) | 1.14 (0.80, 1.62), p=0.46 | 0.08 (-0.14, 0.30), p=0.48 |
| Oxycodone+Celecoxib+APAP | 123 (0.5) | 83 (0.5) | 40 (0.6) | 1.15 (0.79, 1.67), p=0.48 | 0.07 (-0.13, 0.28), p=0.49 |
| Morphine+Tramadol+APAP | 119 (0.5) | 82 (0.5) | 37 (0.5) | 1.07 (0.73, 1.58), p=0.72 | 0.04 (-0.16, 0.24), p=0.72 |
| Oxycodone+Tramadol | 108 (0.5) | 87 (0.5) | 21 (0.3) | 0.57 (0.36, 0.92), p=0.02 | -0.22 (-0.39, -0.05), p=0.01 |
| Hydrocodone+Tramadol | 105 (0.4) | 81 (0.5) | 24 (0.3) | 0.71 (0.45, 1.11), p=0.13 | -0.14 (-0.32, 0.03), p=0.10 |
| Diclofenac+APAP | 99 (0.4) | 53 (0.3) | 46 (0.7) | 2.07 (1.39, 3.06), p<0.01 | 0.34 (0.13, 0.55), p<0.01 |
| Tramadol+Ibuprofen+APAP | 86 (0.4) | 63 (0.4) | 23 (0.3) | 0.87 (0.54, 1.40), p=0.56 | -0.05, (-0.21, 0.11), p=0.55 |
| Celecoxib+APAP | 83 (0.4) | 64 (0.4) | 19 (0.3) | 0.71 (0.42, 1.18), p=0.18 | -0.11 (-0.27, 0.04), p=0.15 |
| Tramadol+Diclofenac+APAP | 83 (0.4) | 50 (0.3) | 33 (0.5) | 1.57 (1.01, 2.44), p=0.04 | 0.17 (-0.01, 0.35), p=0.06 |
| Morphine+Hydrocodone+APAP | 69 (0.3) | 54 (0.3) | 15 (0.2) | 0.66 (0.37, 1.17), p=0.16 | -0.11 (-0.25, 0.03), p=0.12 |
| Other Regimens | 3,382 (14.3) | 2,443 (14.6) | 939 (13.4) | 0.91 (0.85, 0.98), p=0.01 | -1.25 (-2.21, -0.29), p=0.01 |
| Abbreviations: APAP, acetaminophen.  ^1^Onset of COVID 19 Pandemic Considered as March 16, 2020 or later. Pre-pandemic period is from January 1, 2018 to March 15, 2020. Post-pandemic period is from March 16, 2020 to June 30, 2021.  ^2^Risk ratios and percentage point differences (i.e., risk differences) calculated comparing analgesic regimen use after versus before the onset of the COVID-19 pandemic. | | | | | |

**Additional Figure 1.** **Flow diagram of the study population.**

**
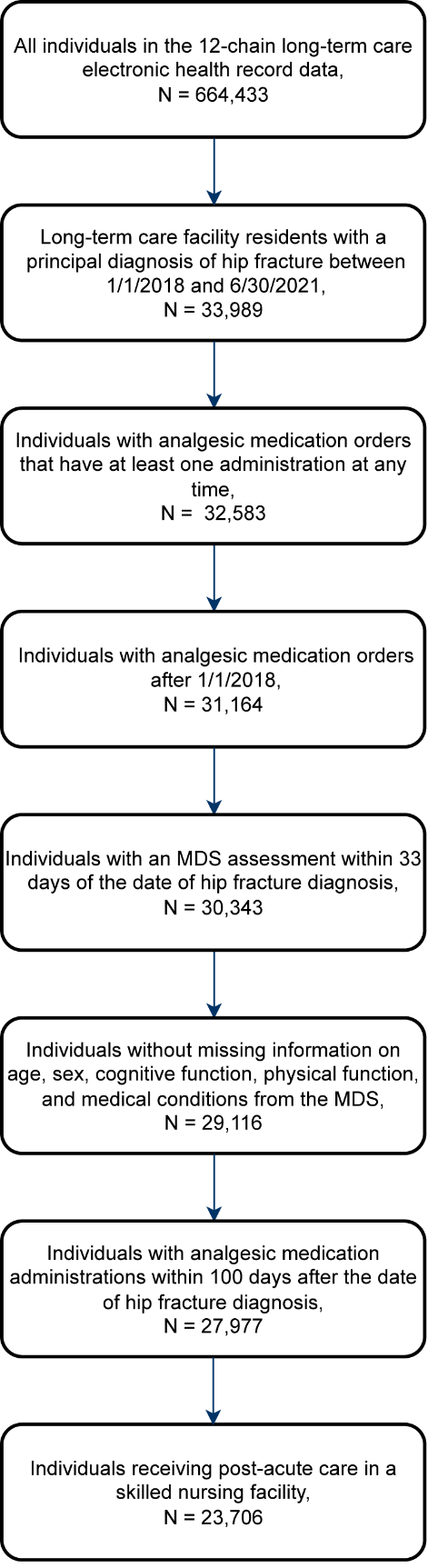
**

**Additional Figure 2. Trends in the use of analgesic medication class-level regimens among patients in U.S. skilled nursing facilities after hip fracture between January 1, 2018 and June 30, 2021 stratified by subgroups of the Gagne Combined Comorbidity Score.** Presents proportion of residents who had at least one administration of the medication class(es) in a given regimen at any point during the quarter of calendar time among patients in each Gagne combined comorbidity score subgroup. Analgesic medication class-level regimens are mutually exclusive categories. The denominator used to calculate the proportion in each quarter is the number of residents who were present in the skilled nursing facilities and received at least one dose of any analgesic in that quarter. Abbreviations: APAP, acetaminophen; NSAIDs, non-steroidal anti-inflammatory drugs.

**
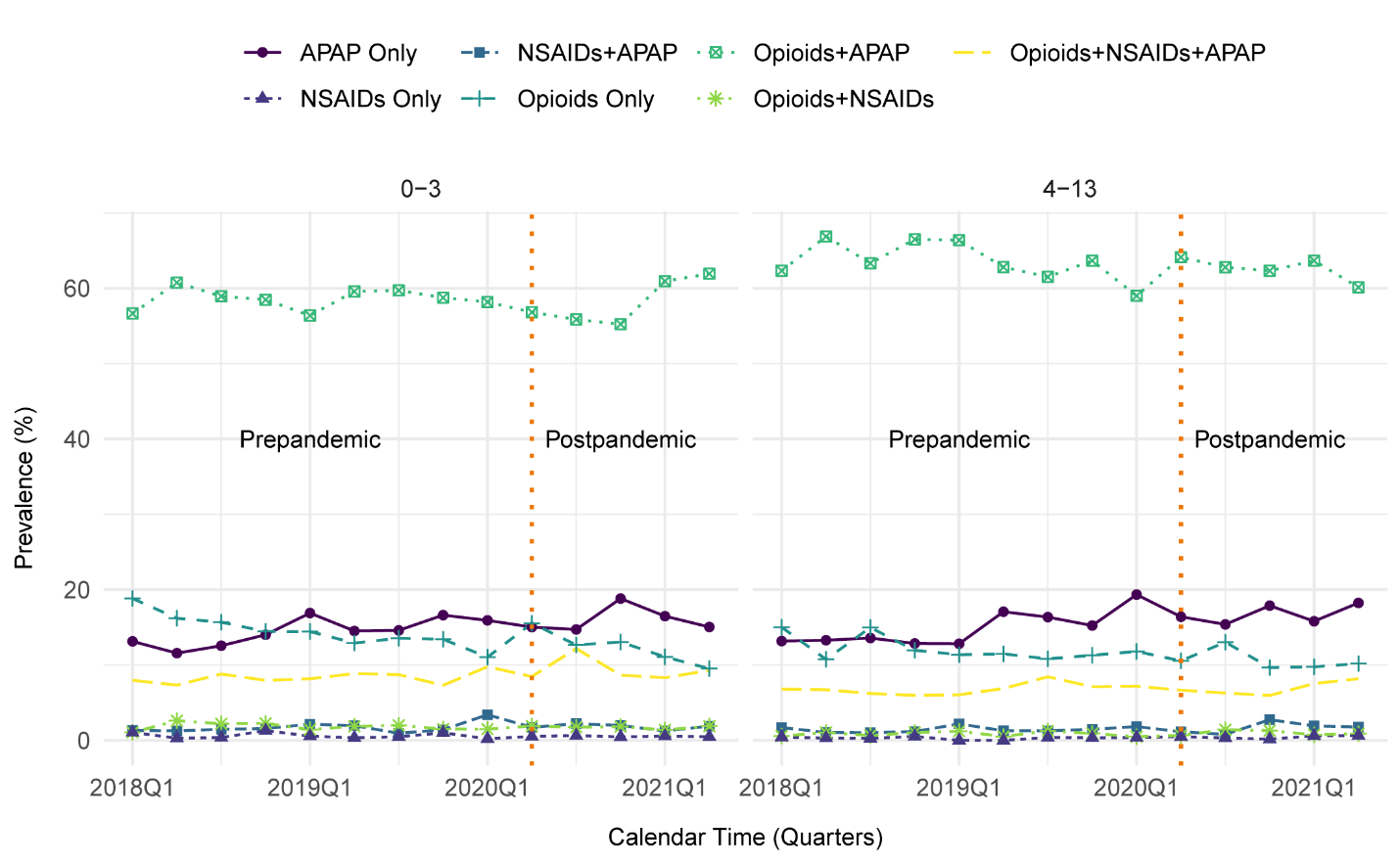
**
